# Factors That Influence Children’s Exits from the Special Supplemental Nutrition Program for Women, Infants, and Children: A Systematic Review

**DOI:** 10.3390/nu15030766

**Published:** 2023-02-02

**Authors:** Karina R. Lora, Leslie Hodges, Cayley Ryan, Michele Ver Ploeg, Joanne Guthrie

**Affiliations:** 1 Department of Exercise and Nutrition Science, The George Washington University, 950 New Hampshire Avenue, Washington, DC 20052, USA; 2 United States Department of Agriculture, Economic Research Service, 1400 Independence Avenue SW, Washington, DC 20250, USA; 3 Department of Sociology and Criminology, Pennsylvania State University, 211 Oswald Tower, State College, PA 16801, USA

**Keywords:** WIC, exits, participation, review, breastfeeding, socioeconomic, stigma, fruit and vegetables

## Abstract

The Special Supplemental Nutrition Program for Women, Infants, and Children (WIC) provides supplemental foods and nutritional education to low-income women and children up to the age of five. Despite evidence that WIC improves diet and nutrition and the nationwide availability of this program, many participants exit WIC before they are no longer eligible for benefits. To date no study has systematically reviewed factors that influence participants’ exits from WIC. The study systematically reviewed the relevant literature to identify factors related to discontinuing participation in WIC before children reach the age of five and 1503 citations were reviewed, 19 articles were read for full text review and eight studies met inclusion criteria. Participants’ higher socioeconomic status, attitudes and behaviors around breastfeeding, having shorter prenatal participation in WIC, administrative barriers, confusion regarding program eligibility, feelings of stigma and embarrassment at the store checkout lines, personal and family challenges, dissatisfaction with insufficient fruit and vegetables benefits and living in suburban areas or in the Southern US were salient factors that influenced WIC exits. These findings will be of interest to policymakers and stakeholders as they consider ways to increase participation and retention through program modernization and innovations.

## 1. Introduction

The Special Supplemental Nutrition Program for Women, Infants and Children (WIC) is a federal nutrition assistance program that provides supplemental foods and nutrition education, including breastfeeding promotion and support, at no cost to pregnant, postpartum and breastfeeding women, infants and young children up to the age of five from lower-income households [1]. To be eligible for WIC, women or children or both must meet income guidelines (as determined by household income ≤ 185% federal poverty level (FPL) [e.g., $51,338 annually for a household size of 4) or participation in Medicaid or another authorized public assistance program), state residency requirements, and be individually determined to be at nutritional risk (medical or diet-based risk) by a health professional [2]. WIC served about 6.2 million participants each month in the fiscal year 2021, including an estimated 43% of all infants in the United States [3].

Previous research has shown that WIC improves diet and nutrition. Longer WIC participation has been associated with higher diet quality among children, measured by total Healthy Eating Index (HEI) scores at age 2 [4] and at age 5 [5]. Additionally, older infants in the program are more likely to eat vegetables, and children in the program were more likely to drink non-fat or low-fat milk compared to their low-income non-participant counterparts [6]. Child WIC participation is also associated with greater consumption of 100% fruit juice and whole grain cereals [7], while household WIC participation is associated with greater purchasing of healthy food groups [7]. The benefits of WIC participation extend from diet and food behaviors to health outcomes including lower risks of prenatal delivery, low birth weight and low infant mortality [7]. Maternal WIC participation is associated with increased child preventative care, increased rate of immunization and higher cognitive scores for children [7].

WIC is a cornerstone of USDA’s efforts to promote nutritional security and by extension, health and, wellbeing. Declining participation is a concern among policymakers and program officials. Between 2009 and 2020 the number of program participants declined each year [8] and in 2019 only 56% of eligible individuals participated in WIC [9]. As part of the American Rescue Plan Act, USDA’s Food and Nutrition Service (FNS), the agency that administers the WIC program, recently received $390 million for outreach, innovation and modernization for WIC. This investment aims to increase WIC’s participation rate through increased enrollment, retention of participants for the full length of their eligibility and an improved participant experience [10].

This paper provides information directly relevant to WIC program modernization efforts by systematically reviewing the existing literature on factors related to discontinuing participation in WIC before children reach the age of five, which we refer to hereafter as WIC exits. From a policy standpoint, a better understanding of the complex process involved in decisions to leave the program can inform programmatic efforts to increase WIC retention. Moreover, studies included in the present review had diverse study designs (i.e., retrospective/secondary data analysis, qualitative) which allowed the current work to provide different perspectives on the topic studied. Across study design and type, we found similar individual and interpersonal factors that influenced participants’ exits. 

Finally, self-selection into WIC is a barrier to understanding the impact of WIC on mothers and their children, since those who exit the program differ systematically from those who remain enrolled in ways that may be linked to diet and health outcomes. To date, many studies have attempted to account for selection bias in analyses of WIC program effects by including socio-demographic characteristics, which differ between the participant and non-participant groups and may contribute to differences in outcomes of interest, as controls or covariates in multivariate regressions [5,11,12]. From a methodological standpoint, this comprehensive account of the characteristics of those who stay in the program and those who exit lays the groundwork for better empirical identification of WIC’s impacts on participants. 

## 2. Materials and Methods

Articles included in this review were extracted from The Cumulative Index to Nursing and Allied Health Literature (CINAHL), Scopus, and PubMed through a systematic review of the literature from December 2021-February 2022. We used the systematic review software Covidence for screening and data extraction and followed the Preferred Reporting Items for Systematic Reviews and Meta-Analyses (PRISMA) format for data reporting. The present review was completed in a systematic, highly rigorous and transparent way to minimize bias. A priori and with the guidance of an experienced medical librarian, we tested key words related to WIC exits found in the Medical Subject Headings Library (http://www.nlm.nih.gov/mesh/) by conducting mock searches to ensure that the final list of terms will be captured in articles that met inclusion criteria. Terms used in the search included a combination of the following words: mother, mothers, caregivers, women, grandparents, WIC, The Special Supplemental Nutrition Program for Women, Infants and Children, food assistance, satisfaction, participation, experience, barrier concern, leave, exit, and retain. Vocabulary and syntax were adjusted across databases. 

The study inclusion criteria focused on factors that influenced exits from the WIC program at or after the participating child turned 1 year old. We excluded studies where only those who continued on WIC were interviewed about their program experience and studies where only data from those who continued were used for analysis. This decision was based on the rationale that the views and characteristics of the caregivers that continued to participate in WIC might have not reflected the views of characteristics of those who exited the program. Further, we did not place limitations on date of publication or study design in the search. Reports were considered, but editorials, commentaries, or meeting abstracts were excluded due to the limited information available. Only articles in the English language and studies that were conducted in the US were included.

Searches were uploaded in Covidence. After all duplicates were removed, three reviewers (K.L., M.P. and C.L.) independently conducted the title and abstract screening to check for inclusion criteria, compared data and resolved discrepancies. Next, two reviewers (K.L. and L.H) independently conducted full text review of the articles that were considered relevant. The reference lists of these articles were reviewed to include relevant articles not initially part of the title and abstract screening, resulting in the addition of one study for full text screening. This step minimized risk of bias due to potentially missing results in the initial synthesis. The Review Board determined that the study did not require review for human subject research.

## 3. Results

**Figure 1:**
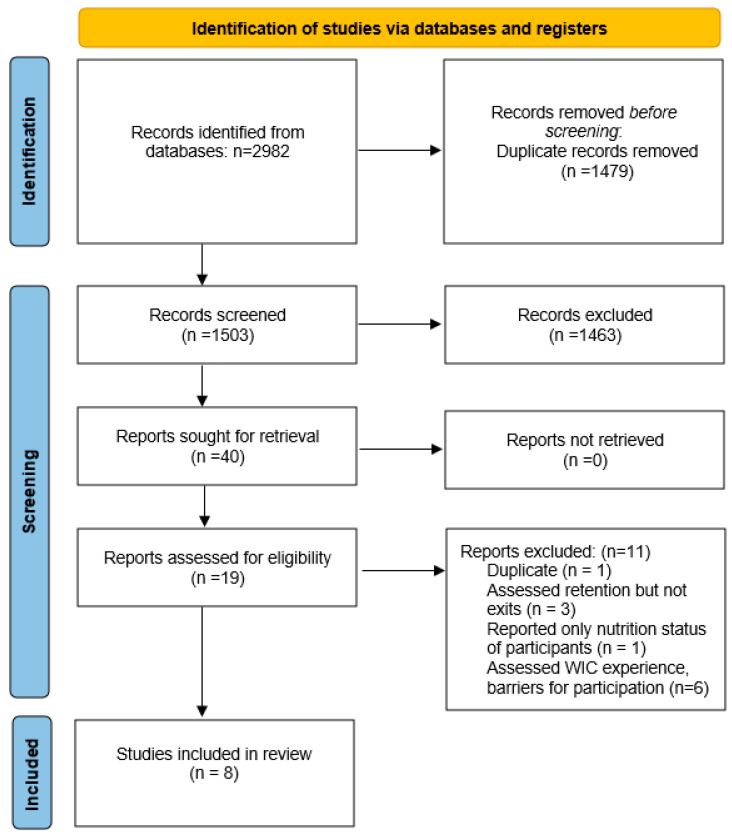
PRISMA flow diagram for the systematic review.

In total 1503 articles’ titles and abstracts were initially screened, 19 articles were read for full text review (including the one additional study) and 11 were excluded because they did not meet inclusion criteria, resulting in eight articles used for data extraction [13,14,15,16,17,18,19,20] (Figure 1). Six studies were quantitative, one was qualitative and one was a mixed methods study. Four of the six quantitative studies were retrospective and used secondary data and two were survey-based cross-sectional studies. The qualitative study used semi-structured interviews, whereas the mixed methods study used focus groups, interviews, and direct observations. Children’s caregivers provided data for themselves and for the child. In the present study, children’s caregivers are referred to as participants (Table 1).

### 3.1. Criteria Used to Assess WIC Exits 

Studies used diverse criteria to assess child exits from the WIC program. This included participant reports of voluntary exits from the program obtained from survey responses (e.g., (caregiver left the program when the child turned 1 year old or chose to leave the program for reason unrelated to ineligibility), and participants who initially reported receiving WIC but indicated later not being part of the program in longitudinal studies (e.g., children in households that reported receiving WIC in month m of the survey and then did not receive WIC in later survey months; participants who were initially enrolled in WIC but then reported not participating in the program when the child was 14 months old). Studies also used administrative records to assess child exits from the WIC program. This included records indicating that the participant was terminated from WIC due to failure to attend appointments with food benefit issuance for 60 days (non-participation), and child identification number absent from monthly participation record or a new certification date was not present in two consecutive months.

### 3.2. Demographic Characteristics of Study Samples

Eight studies reviewed reported race and/or ethnicity of their study samples or provided a measure that could serve as a proxy for race and/or ethnicity. In five studies a majority of the study sample identified as non-Hispanic White [14,16,20] or White of any ethnicity [13,17]. In one study a majority of the study sample identified as non-Hispanic Black [15]. In two studies a majority of the study sample identified as Hispanic [13,18]. In two studies a majority of the study sample preferred English language [17,19]. Three studies reported the age of mothers/caretakers in their study samples. In these studies, the majority of mothers/caretakers were younger than 29 years [16,18] or had a mean age of 29 years [20]. Two studies reported on the citizenship or nativity of mothers/caretakers in their study samples. In one study, 90% of mothers/caretakers were foreign born [18]. In another study, 78% of mothers/caretakers were U.S. citizens [16].

### 3.3. Main Findings

Studies that reported participants’ exits via information obtained from quantitative and qualitative studies indicated that administrative barriers such as difficulty scheduling, rescheduling and recertifying appointments, an extensive wait time during check pick up (data collected when WIC benefits were provided via paper vouchers or “checks” rather than current use of Electronic Benefit Transfer cards), and needing to comply with redundant clinical requirements were factors that influenced exits [13,17,20] (Table 2). Participants’ confusion about program eligibility, perceptions that they were taking benefits that others were more deserving of, transportation problems to reach the clinics, childcare challenges, and the existence of family illness, or job conflicts were also reported barriers [13,14,16,17,18]. Dissatisfaction with the dollar amounts of the cash-value benefit for fruit and vegetable purchases (CVB) in the food packages as well as a lack of provision of more culturally appropriate food choices were also mentioned as reasons for exits [13]. The dissatisfaction with the CVB pre-dated the temporary increases in the CVB amount during the COVID-19 pandemic. Feelings of embarrassment at store checkouts due to item mislabeling (i.e., product labeled as WIC eligible, but it was not) was also a factor that influenced exits from the program [13,17] (Table 3). While the use of EBT cards—implemented in 2010—and smartphone apps have improved the shopping experience, embarrassment at checkout still persists. In addition, general stigma revolving around being a WIC program participant was also a factor that influenced exits from the program. 

Studies that used administrative records to assess child exits from the WIC program reported that in general participants with higher socioeconomic status (SES) were more likely to exit WIC [14,16,19]. For instance, participants with higher incomes, higher educational attainment (high school degree or higher), higher rates of maternal employment or participants who did not receive SNAP were more likely to exit WIC [14,16,19]. Receiving Medicaid benefits was also reported as a factor associated with increased likelihood of leaving [14,19]. However, one study reported that participants who received Medicaid for their children were less likely to exit WIC [18]. Further, one study used in the present review reported on participants with military health care and found that those who received health care from a health maintenance organization were more likely to exit WIC than participants who received healthcare from a private physician [15]. Area of residence and household geographical location were also related to exits from WIC. Participants living in suburban areas were more likely to exit WIC than urban residents [15]. Participants living in the Southern region of the U.S. and in small towns were also more likely to exit WIC [16]. Breastfeeding or breastfeeding intentions were factors associated with WIC participation. For instance, one study reported that, compared to child participants that recertified for WIC by 14 months of age, participants who did not recertify after their child turned 1 year of age were less likely to fully breastfeed and less likely to have prenatal intention to breastfeed [19] Another study reported that participants who had higher rates of never breastfeeding or breastfed for less than 6 months were likely to exit WIC [16]. Other factors such as enrolling the child in WIC at a later age (7–12 months vs. 6 months or younger) [15], having shorter prenatal participation (2.47 months vs. 3.08 months) [16,19] and beliefs about not being eligible for WIC benefits [14] also influenced program exits.

## 4. Discussion

WIC participation is at its highest in infancy; when infants transition to the child package, participation drops despite considerable evidence that WIC participation is associated with better childhood diets. The evidence presented in this systematic review shows that several individual and interpersonal factors influenced participants’ exits from WIC at this critical juncture. Participants’ higher socioeconomic status; attitudes and behaviors around breastfeeding, such as low intentions to breastfeed during the prenatal period, never breastfeeding or breastfeeding for less than 6 months; and having shorter prenatal participation in WIC were factors related to WIC exits [14,16,19]. Further, administrative barriers, confusion about program eligibility, feelings of stigma and embarrassment at the store checkout lines, personal (i.e., transportation) and family challenges, dissatisfaction with insufficient fruit and vegetables benefits (prior to the temporary CVB increase during the pandemic and the proposed rule released in November 2022 that would make these increases permanent), and living in suburban areas or in the Southern US were also factors [13,15,17,18,20]. 

The factor most commonly associated with exits from the WIC program among studies in this review was a participant’s higher socioeconomic status within the economic range eligible for WIC. Parents/caretakers of children with higher incomes, higher rates of employment, higher education (completed at least high school) and higher household savings were less likely to continue receiving WIC benefits. While this may suggest that children who continue receiving WIC services are from families with higher economic needs and thus less likely to be income ineligible for WIC, it is also possible that infant/children that exit WIC remain eligible and could continue to benefit from the program if they reenrolled. Gundersen (2005) indicated that infants who left WIC were from families that had incomes below the poverty line, received federal food assistance and almost all remained eligible for WIC [14]. Similarly, Jacknowitz and Tiehen (2009) indicated that late entrants and early leavers of the WIC program still exhibited economic needs [16]. Program exits by eligible participants that receive federal food (SNAP) and medical (i.e., Medicaid) assistance while they still qualify for benefits are common [21]. For instance, Gray and O’Leary (2019) suggested that about half of participant households receiving SNAP benefits that exit within one year of entry are actually eligible on their one-year anniversary [22]. Further, two studies used in the present review found that participants who exited WIC were less likely to continue to participate in Medicaid or SNAP [14,19]. One study reported that women who received Medicaid for their child were less likely to exit WIC compared to those who had not received Medicaid for their children [18]. 

Low adherence to breastfeeding recommendations was also associated with exits from WIC in childhood. Participants who reported never breastfeeding or breastfeeding for less than 6 months were more likely to exit WIC [16]. Further, mothers who did not recertify their children in WIC after the child reached 1 year of age were less likely to have prenatal intention to breastfeed than mothers who recertified their children in WIC [19]. These findings suggest that efforts to increase breastfeeding intention and duration among WIC participants may also keep participants engaged in WIC [19]. It is also possible that participants that breastfeed their infants are different from participants who formula feed their children in a way that keeps them engaged in WIC. Breastfeeding is a priority in the WIC program. Program participation data of the number of partially and fully breastfed infants for each WIC state and local agency for year 2021 indicate that the infant breastfeeding rate (fully and partially breastfeeding) was 34.3%, a 0.4% increase from the previous year [23]. Women who fully or mostly breastfeed receive food packages for themselves; some iron-fortified formula is provided for infants of partially breastfeeding women, whereas for women who do not fully breastfeed, WIC provides larger amounts of iron-fortified infant formula [24]. When infants reach 6 months, infants of breastfeeding and formula feeding women receive infant cereal baby fruits and vegetables; infants of breastfeeding mothers also receive baby food meat. At age 1 year, WIC provides packages suitable for young children. Multiple studies reported that participants who never breastfeed or breastfeed for less than 6 months [16] or had a low intention to breastfeed left the program [19]. Future studies could assess whether participants who formula feed might not value food incentives provided to children one year and above as much as participants who breastfeed, or if other individual factors play a role in the decision to discontinue participating in WIC. For instance, mothers participating in WIC who formula fed their infants discussed feeling judged for not breastfeeding, perceiving WIC as a formula provider, and perceiving difficulty receiving the desired amount of formula from WIC needed to feed their ≥ 6 months infant. Mothers reported that these experiences affected motivations to recertify when their children were 1 year old [25].

WIC, like other food and social assistance programs, requires beneficiaries to periodically reverify eligibility. Before the COVID-19 pandemic, only about half of all eligible families received WIC, with many eligible families leaving the program due to the complexity of the recertification process [26]. While the program had adopted some streamlined certification practices such as appointment reminders or reviews of electronic documents during appointments, online appointment scheduling or electronic submissions of documents for recertification were not widely used practices in managing operations prior to the pandemic. The passage of the Families First Coronavirus Response Act [27] allowed WIC to implement COVID-19 waivers to temporarily address a number of administrative barriers that participants reported as factors for program exits. Waivers implemented included remote benefit issuance, waivers for in-person enrollment, extension of waivers and extended recertification [28]. There is some evidence that these resulted in WIC participation increases. Comparing February 2020 to February 2021, WIC participation increased from 6.1 million to 6.2 million participants, an increase of 2.1% [29,30]. It is important to keep in mind that the flexibilities in the administrative certification and recertification processes put in place in response to the COVID-19 pandemic were temporary and many WIC sites have returned to business-as-usual approaches to providing services. It is also important to keep in mind that the current evidence on the effectiveness of these waivers for increasing participation does not address whether the waivers increased retention among those with highest risks of exit from the program (i.e., groups identified in this systematic review).

The ARPA modernization and innovation funds and Child Nutrition Reauthorization provide important opportunities for making permanent changes to the WIC program to increase participation and retention. For example, one approach currently being considered to increase caseload and reduce exits from WIC is opening clinics late during the day or offering Saturday clinics [31]. This approach would be in line with findings from our review that participants that exited WIC reported transportation problems and being challenged by job time conflicts [18,20]. Technology modernization may increase use of strategies such as online “chatbots” that can answer common questions about eligibility and simplify appointment scheduling reducing administrative barriers to participation [32].

Feelings of embarrassment at checkout due to in-store item mislabeling of foods and stigma were factors related to exits from the program. WIC participants’ feelings of feeling stigmatized from community members and/or at store checkout lines have been previously reported [33,34]. A qualitative study with pregnant women and mothers participating in WIC reported that the most pervasive barrier to participation was social stigma [35]. Participants discussed that the checkout experience evoked feelings of anxiety and embarrassment [35]. The most recent child nutrition reauthorization, The Healthy, Hunger-Free Kids Act of 2010, mandated that by 2020 all WIC State agencies use Electronic Benefits Transfer (EBT) to disburse benefits. Use of EBT has been suggested as potentially reducing in-store stigma possibly by reducing the time associated with transaction at the store checkout line and making it difficult to identify beneficiaries [33,34,36]. It has been suggested that creating a better shopping experience, such as having a section for WIC items or clearer labeling of WIC-approved items in the store or improving in-store education, may improve the retail experience and that these strategies may decrease program exit [34]. USDA is also piloting programs that allow for participants to shop for WIC foods online, which may also help to reduce factors that influenced exits such as feelings of embarrassment and social stigma [37].

In addition to limited selection of WIC foods available at certain stores being cited as barriers to continued participation in WIC [38], dissatisfaction with insufficient fruits and vegetables has also emerged as a factor related to exits [13,16]. Most items in the WIC food packages are redeemable for specified quantities—for example, 1 dozen eggs or 4 gallons of milk—and the amounts provided to recipients do not vary with household income. However, since 2009, food packages have also included a cash-value benefit (CVB), which is a fixed-dollar-amount benefit that can be used to purchase a variety of fruit and vegetables of the participant’s choosing. The value of the CVB was temporarily increased during the COVID-19 pandemic from $9 (adults) and $11 (children) to $24 for child participants, $43 for pregnant and postpartum women participants and $47 for fully and partially breastfeeding women participants. Higher CVB allotments have been found to increase WIC participants’ purchasing and consumption of fruits and vegetables, increase the frequency of their shopping occasions, and enhanced their dietary variety [39]. The proposed rule on WIC food package revisions released by USDA, FNS in November 2022 would make these increases permanent [40] providing participants with up to four times the amount of fruit and vegetables they would otherwise receive, a greater variety of fruit and vegetables to choose from, and adjust the quantity of fruit juice to reflect nutrition recommendations from the National Academies of Science, Engineering, and Medicine and the Dietary Guidelines for Americans, 2020–2025 [41].

Finally, living in suburban areas or in the Southern US also emerged as a factor associated with early exits from the program. In southeastern states such as Mississippi, Arkansas, Kentucky, Louisiana, South Carolina and Alabama over 60% of all infants participate in WIC [42]. Two studies, one conducted in South Carolina and the other in Mississippi, found that WIC participants were less likely to initiate breastfeeding compared to income-eligible non-WIC participants, and income-ineligible non-WIC participants [43]. They also found that WIC participation was not associated with breastfeeding duration in White and Black participating women in these states [44]. The present review found who participants that exited WIC had low breastfeeding intentions and rates. It is possible that while WIC participation is high among southern participants, their low breastfeeding initiation or duration behaviors may influence exits after the first months of children’s lives. Further, WIC state policies have been suggested to play a role in participation decisions for households residing in the southern region of the US [16]. Noteworthy is that, according to WIC data, the states with the largest numbers of fully formula fed infants are Alabama (89.0%), Arkansas (86.4%), Louisiana (84.5%) and Mississippi (85.4%), all located in the southern region of the US [23]. In considering ways to promote continued WIC participation, future work may want to consider the interplay among participants’ time of exit from the WIC program, breastfeeding duration and use of formula.

The study had multiple strengths. This is the first systematic review to report factors that influence participants exits from the WIC program. Studies included in the present review had diverse study designs (i.e., retrospective/secondary data analysis, qualitative) which allowed the current work to provide different perspectives on the topic studied. We did not restrict publication year of the studies included in the review. This allowed us to capture factors related to WIC exits in the context of changes the WIC program has undergone through the years. In addition, the methodology followed in the present systematic review minimized risk of bias. Study limitations include a lack of consistency in the factors investigated related to exits from the WIC program in all studies included in the present review. Socioeconomic status was identified in more studies than other factors; however, other factors such as household location in the Southern region of the U.S, embarrassment at checkout due to item mislabeling, or stigma were only assessed in three different studies. Further, some studies had larger, more nationally representative samples than others making generalizability of each finding challenging. Studies addressing these limitations may add to our understanding of factors influencing WIC exits. Further, there is a need for future research to examine how and to what extent the factors related to exits from WIC found in the present review change over time and in response to different policy and economic contexts, and to continue to monitor to what extent these factors remain relevant in the current policy and economic context.

## 5. Conclusions

In conclusion, understanding the complex factors that influence exits from the WIC program opens opportunities for engaging participants and addressing barriers to continued participation. The studies we reviewed suggest that streamlining certification and recertification processes, continuing the promoting and advocating for breastfeeding adherence (particularly during the first 6 months of a child’s life) and providing positive client-staff interactions in which WIC participants feel supported all have the potential to reduce participants’ exits. The effectiveness of strategies addressing these factors has not been tested. In terms of messaging about WIC, our review findings suggest that communication and outreach with current and prospective participants about eligibility, particularly those closer to the income eligibility threshold, is another potential method to increase participation among those eligible. Our findings also reinforce the importance of modernization such as the use of electronic benefits and mobile apps. In particular, mobile apps offer opportunities to improve WIC participants’ shopping experience and increase redemption rates of food packages [45] and may reduce stigma [33]. Further, virtual interventions that are single faceted and focus on direct and simple strategies (e.g., nutrition education or how to transition from paper vouchers to EBT) have been found to be the most successful at increasing enrollment and participation rates in WIC [46]. Future studies should consider examining similar factors found to be related to exits from the WIC program in the present review among diverse groups to elucidate characteristics (i.e., language spoken) that are unique to racial/ethnic families. Such studies could advance the USDA FNS goal of reducing disparities in participation and outcomes among WIC participants.

## Figures and Tables

**Table 1:** Participants’ characteristics and factors that influence participants exiting the USDA Special Supplemental Nutrition Program for Women, Infants, and Children (WIC).

Authors	Design/Data Collection/Data Analysis/Location	Participants	Criteria Used to Assess WIC Exit	Demographic Characteristics of Study Sample ^a^	Main Findings
Hammad T et al., 1997 [15]	Retrospective/secondary data analysis of administrative records/Maryland, US	Caregivers of infants and children up to 3 years old (*n* = 12,749)	Child identification number absent from monthly participation record, or a new certification date was not present on monthly participation record for two consecutive months	40% were non-Hispanic White, 53% were non-Hispanic Black, 5% were Hispanic of any race; 2% were non-Hispanic of another race	Children who enrolled in WIC at 7–12 months were more likely to exit WIC within a year than children who enrolled in WIC at ages 6 months and younger (RR ^b^ = 1.34; 95% CI ^c^ = 1.26, 1.43) Participants with military health care and those that received health care from a health maintenance organization were more likely to exit WIC than participants that received healthcare from a private physician (RR = 1.32; 95% CI = 1.21, 1.44, and RR = 1.26; 95% CI = 1.16, 1.37, respectively) Suburban residents were more likely (RR = 1.06; 95% CI = 1.02, 1.11) while rural residents were less likely (RR = 0.85; 95% CI = 0.81, 0.90) to exit WIC than urban residents Families with five or more members were less likely to exit WIC than those with two members (RR = 0.94; 95% CI = 0.89, 0.99)
Rosenberg T et al., 2003 [18]	Cross sectional/structured questionnaire with close and open-ended questions/New York, US	Former WIC participants who had withdrawn from the program (“leavers,” n = 188) and current WIC clients (“clients,” *n*= 280)	WIC records contained an entry of “void” or “void unclaimed” for check registry meaning that the participant did not pick up or redeem WIC food package benefits	90% identified as Hispanic. 51.6% were between 15–29 years, 48.4% were ≥ 30 years. 90% were foreign born	Women who had received WIC themselves were more likely to exit the program compared to women who had not received WIC themselves (AOR ^d^ = 1.90; 95% confidence interval [CI] = 1.04, 3.46; *p* < 0.05) Women who received Medicaid for their child were less likely to exit WIC compared to those who had not received Medicaid for their children (AOR = 0.50; 95% CI = 0.30, 0.84; *p* < 0.01). Other factors associated with exits from WIC included: Transportation problems (AOR = 2.00; 95% CI = 1.08, 3.72; *p* < 0.05), family illnesses (AOR = 2.68; 95% CI = 1.48, 4.85; *p* < 0.01), and job conflicts (AOR = 1.87; 95% CI = 0.30, 0.84; *p* < 0.05)
Woelfel M et al., 2004 [20]	Cross sectional/survey (based on literature review, focus groups with caretakers of WIC participants, and expert panel input)/New York, US	Parents/caretakers of infants and children (*n* = 3167) on WIC	Did not redeem or pick up WIC food package benefits (paper checks)	50% were non-Hispanic White, 30% were non-Hispanic Black, 15% were Hispanic of any race. The mean age of parent/caretaker was 29 years	Those that exited WIC reported difficulty rescheduling appointments (*n* = 309), difficulty recertifying (*n* = 267), or having to wait too long when picking up checks
Gundersen C, 2005 [14]	Retrospective/secondary data analysis from the 1996 panel of the Survey of Income and Program Participation/nationally representative, US	Infants and children income-eligible for WIC in month 12 of the 1996 panel of the Survey of Income and Program Participation (*n* = 3471)	WIC-eligible infants and children who received WIC in month 12 of the survey and then did not receive WIC in later survey months	48% were non-Hispanic White, 24% were non-Hispanic Black, 24% were Hispanic of any race; 4% were non-Hispanic of another race or unknown	On average, families that exited WIC had incomes higher than those who continued receiving WIC (average monthly income = $2300 per month), were less likely to continue to participate in SNAP ^e^ (66%) and Medicaid (60%), and were less likely to be income-eligible for WIC ^f^
Jacknowitz A and Tiehen L, 2009 [16]	Retrospective/secondary data analysis from the 9-month (*n* = 10,700) and 2-year (*n* = 9850) waves of the Early Childhood Longitudinal Study Birth Cohort/national, US	WIC participants (*n* = 4050)	Left the program (no longer reported WIC participation) when the child turned 1 year old	39% non-Hispanic White; 22% were non-Hispanic Black; 34% were Hispanic of any race; 2% were non-Hispanic Asian, and 3% were non-Hispanic of another race. 13% of were <20 years old, 38% were between 20–24 years, 26% were between 25–29 years, 15% were between 30–34 years, and 9% were over the age of 35. 78% were U.S. citizens	Factors associated with exits from WIC included: higher household income, higher rates of maternal employment before and after the survey child’s birth, more household savings and assets, vehicle ownership, post- natal participation in other assistance programs, higher levels of maternal education, higher rates of maternal smoking, higher rates of never breastfeeding or breastfeeding for less than 6 months, and beliefs about not being eligible for WIC benefits, household location in the Southern region of the U.S., household location in towns with population <2500
Panzera A et al., 2017 [17]	Cross sectional/focus groups, interviews, and direct observations at WIC clinic/Kentucky, US	WIC participants/former WIC participants. Focus groups (*n* = 19), individual interviews (*n* = 1), and observed at WIC clinic (n = 6)	Terminated from WIC due to non-participation (failure to attend appointments with food benefit issuance for 60 days)	Focus groups participants age range was 19–51 years, majority White, English speakers	Difficulty scheduling appointments, lack of transportation, childcare challenges, long waits, confusion about program eligibility criteria and procedures, negative interactions with program staff, problems redeeming program benefits, and stigma.
Whaley et al., 2017 [19]	Retrospective/secondary data analysis of administrative records/Los Angeles-Orange-San Bernardino counties, US	Infants receiving WIC benefits for at least 1 month between age 7 and 12 months (*n* = 9632)	Responded “no” to recertification question by when child was 14 months	67.4% preferred English language, used by authors as a proxy for race and ethnicity	The non-recertified group was statistically significantly different from the recertified/participant group in the following metrics: Less likely to fully breastfeed (11.1% vs. 16.5%; *p* < 0.001); less likely to have prenatal intention to breastfeed (59.1% vs. 76.8%, *p* < 0.001); more likely to have missed at least one month of benefits between 6–12 months (48.4% vs. 14.4%; *p* < 0.001) and redeemed <75% of benefits (41.7% vs. 22.9%; *p* < 0.001) Had longer prenatal WIC participation (4.85 months vs. 6.23 months; *p* < 0.001); were less likely to have other family members receiving WIC (1.36 average family members vs. 1.41; *p* < 0.01); had greater household income (71.3% at or below 100% FPL vs. 76.4%; *p* < 0.001) ^g^; were more educated (67.6% with high school degree or higher vs. 60.1; *p* < 0.001); prefer English (83.2% vs. 63.9%; *p* < 0.001); and less likely to participate in Medicaid (66.9% vs. 75.5%; *p* < 0.001)
Gago C et al., 2022 [13]	Cross sectional/semi structured interviews/Massachusetts, US	Caregivers of WIC eligible children under the age of 5 years who were currently enrolled in WIC or children who were 6–24 months before the date of the interview (total = 37, current = 18, early leavers = 17)	Chose to leave WIC for reasons other than ineligibility	47% were White, 18% were Black, 35% were of another race 63% were Hispanic 29% were between 18 and 29 years; 32% between the ages of 30–34 and 38% were 35–44 years	Families reported exiting WIC because of: Insufficient fruit and vegetable benefits Food benefits’ inflexibility to allergies, cultural appropriateness, and individual preferences Embarrassment at checkout due to in-store item mislabeling Administrative barriers, including lack of clarity in requirements for certification; challenges rescheduling appointments; long wait times; challenges with transportation to and from appointments; and clinical requirements they felt were unnecessary, redundant, and/or burdensome Perceptions that they were taking benefits that others would need more

^a^ Demographic characteristics include race and ethnicity, mother or caretaker’s age, and mother’s citizenship (U.S. citizen or not) or nativity (U.S. born or not) if reported. ^b^ RR: Relative Risk. ^c^ CI: Confidence Interval. ^d^ AOR: Adjusted Odds Ratio. ^e^ SNAP: Supplemental Nutrition Assistance Program. ^f^ Those that left WIC were less likely to be income-eligible for WIC than those that remained on the program and more likely to be income-eligible for WIC than those who never participated. ^g^ FPL: Federal Poverty Level.

**Table 2:** Participant characteristics associated with exiting the USDA Special Supplemental Nutrition Program for Women, Infants, and Children (WIC).

Demographic Factors	
*Race & ethnicity*	Non-Hispanic White [16] Non-Hispanic of any race [13]
*Language*	Preferred English language [19]
*Child’ age*	Later enrollment in WIC during infancy [15]
*Parent’s age*	Less than 30 years [13,16,18]
*Family size*	Smaller families [15]
Socioeconomic factors	
*Educational attainment*	High school completion [13,18,19] More than high school [16,19]
*Income & assets*	Higher household income [14,16,19] Household savings and assets [16] Vehicle ownership [16]
*Health insurance*	Military healthcare [15] Healthcare from health maintenance organization [15] Less likely to receive Medicaid [14,18,19]
*Means-tested programs*	Less likely to participate in SNAP [13,14,18] Less likely to participate in TANF [18]
Feeding Practices	
*Breastfeeding*	Less likely to fully breastfeed [19] Less likely to report prenatal intention to breastfeed [19] Never breastfeeding [16] Breastfeeding for less than 6 months [16]
Experience with WIC	
*Prior*	Mother received WIC themselves [18]
*Current*	Missed at least one month of benefits between 6–12 months [19] Redeemed less than 75% of benefits [19] Longer prenatal participation [19]

**Table 3:** Participants’ reported reasons for exiting the USDA Special Supplemental Nutrition Program for Women, Infants, and Children (WIC).

Ineligibility
Income ineligibility [13,14,16,17] Eligible but on waitlist due to lack of program funds [16]
Participant Experience
*Administrative factors*
Difficulties recertifying and rescheduling appointments [13,17,20] Long waiting lines to pick up checks [13,20] Negative interactions with program staff [17] Lack of clarity in requirements for certification and redundant requirements [13,16] Benefits not worth the time required to recertify [16]
*Food benefits*
Individual preference [13] Cultural appropriateness [13] Allergies [13] No longer needed food benefits [16]
*Societal expectations*
Stigma [17] Embarrassment [13]
Work & Family
Transportation problems [13,16,17,18] Family illnesses [18] Job conflicts [18]

## Data Availability

Findings based on publicly available literature as described in Table 1.

## References

[1] National Archives and Records Administration Title 7, Part 246. Special Supplemental Nutrition Program for Women, Infants and Children. https://www.govinfo.gov/app/details/CFR-2022-title7-vol4/CFR-2022-title7-vol4-part246.

[2] WIC Frequently Asked Questions (FAQs). https://www.fns.usda.gov/wic/frequently-asked-questions.

[3] WIC Program. https://www.ers.usda.gov/topics/food-nutrition-assistance/wic-program/#:~:text=This%20amounts%20to%20the%20cost,to%20apply%20for%20the%20program.

[4] Weinfield N.S., Borger C., Au L.E., Whaley S.E., Berman D., Ritchie L.D. (2020). Longer participation in WIC Is associated with better diet quality in 24-month-old children. J. Acad. Nutr. Diet..

[5] Anderson C.E., Martinez C.E., Ritchie L.D., Paolicelli C., Reat A., Borger C., Whaley S.E. (2022). Longer Special Supplemental Nutrition Program for Women, Infants, and Children (WIC) participation duration is associated with higher diet quality at age 5 years. J. Nutr..

[6] Guthrie J.F., Anater A.S., Hampton J.C., Catellier D.J., Eldridge A.L., Johnson W.L., Quann E.E. (2020). The Special Supplemental Nutrition Program for Women, Infants, and Children is associated with several changes in nutrient intakes and food consumption patterns of participating infants and young children, 2008 compared with 2016. J. Nutr..

[7] Caulfield L.E., Bennett W.L., Gross S.M., Hurley K.M., Ogunwole S.M., Venkataramani M., Lerman J.L., Zhang A., Sharma R., Bass E.B. (2022). Maternal and Child Outcomes Associated With the Special Supplemental Nutrition Program for Women, Infants, and Children (WIC).

[8] Toossi S., Jones J.W., Hodges L. (2021). The Food and Nutrition Assistance Landscape: Fiscal Year 2020 Annual Report. https://www.ers.usda.gov/webdocs/publications/101909/eib-227.pdf?v=5724.2.

[9] Farson Gray K.B.-C.E., Giannarelli L., Johnson P. (2022). Estimates of WIC Eligibility and WIC Program Reach in 2019. https://www.fns.usda.gov/wic/national-state-level-estimates-eligibility-program-reach-2019.

[10] Modernizing WIC. https://www.fns.usda.gov/infographic/modernizing-wic.

[11] Borger C., Paolicelli C., Sun B., Zimmerman T.P., Dixit-Joshi S. (2022). Duration of WIC Participation and Early Feeding Practices Are Associated With Meeting the Added Sugars Recommendation at Age 3 Years. J. Nutr. Educ. Behav..

[12] Borger C., Paolicelli C.P., Sun B. (2022). Duration of Special Supplemental Nutrition Program for Women, Infants, and Children (WIC) Participation is Associated With Children’s Diet Quality at Age 3 Years. Am. J. Prev. Med..

[13] Gago C.M., Wynne J.O., Moore M.J., Cantu-Aldana A., Vercammen K., Zatz L.Y., May K., Andrade T., Mendoza T., Stone S.L. (2022). Caregiver perspectives on underutilization of WIC: A qualitative study. Pediatrics.

[14] Gundersen C. (2005). A dynamic analysis of the well-being of WIC recipients and eligible non-recipients. Child. Youth Serv. Rev..

[15] Hammad T.A., Havas S., Damron D., Langenberg P. (1997). Withdrawal rates for infants and children participating in WIC in Maryland. J. Am. Diet. Assoc..

[16] Jacknowitz A., Tiehen L. (2009). Transitions into and out of the WIC program: A cause for concern?. Soc. Serv. Rev..

[17] Panzera A.D., Bryant C.A., Hawkins F., Goff R., Napier A., Schneider T., Kirby R.S., Coulter M.L., Sappenfield W.M., Baldwin J. (2017). Mapping a WIC mother’s journey: A preliminary analysis. Soc. Mark. Q..

[18] Rosenberg T.J., Alperen J.K., Chiasson M.A. (2003). Why do WIC participants fail to pick up their checks? An urban study in the wake of welfare reform. Am. J. Public Health.

[19] Whaley S.E., Whaley M., Au L.E., Gurzo K., Ritchie L.D. (2017). Breastfeeding is associated with higher retention in WIC after age 1. J. Nutr. Educ. Behav..

[20] Woelfel M.L., Abusabha R., Pruzek R., Stratton H., Chen S.G., Edmunds L.S. (2004). Barriers to the use of WIC services. J. Am. Diet. Assoc..

[21] Gray C. (2018). Why Leave Benefits on the Table? Evidence from SNAP. Soc. Mark. Q..

[22] Gray C., O’Leary C. (2019). Increasing beneficiary retention in food assistance programs. Policy Res. Briefs.

[23] Breastfeeding Data Local Agency Report. FY 2021 Report. https://www.fns.usda.gov/wic/wic-breastfeeding-data-local-agency-report.

[24] WIC Food Packages—Maximum Monthly Allowances. https://www.fns.usda.gov/wic/wic-food-packages-maximum-monthly-allowances.

[25] Almeida R., Alvarez Gutierrez S., Whaley S.E., Ventura A.K. (2020). A qualitative study of breastfeeding and formula-feeding mothers’ perceptions of and experiences in WIC. J. Nutr. Educ. Behav..

[26] The Impact of In-Person Benefit Reloading on WIC Participation During the COVID-19 Pandemic. https://policylab.chop.edu/blog/impact-person-benefit-reloading-wic-participation-during-covid-19-pandemic.

[27] Congress (2020). Gov. H.R.6201—Families First Coronavirus Response Act. https://www.congress.gov/bill/116th-congress/house-bill/6201.

[28] WIC COVID-19 Waivers. https://www.fns.usda.gov/programs/fns-disaster-assistance/fns-responds-covid-19/wic-covid-19-waivers.

[29] Food Research and Action Center (2021). One Year of WIC During COVID-19: Waivers are Vital to Participation and Benefit Redemption. https://frac.org/wp-content/uploads/One-Year-of-WIC-During-COVID-19.pdf.

[30] The Network for Public Health Law. WIC: Lessons Learned from COVID-19. https://www.networkforphl.org/wp-content/uploads/2022/05/WIC-COVID-Issue-Brief-Final.pdf.

[31] Bess S., Odoms-Young A., Uesugi K., Brooks T. Retention of Participants Through the First Five Years.

[32] Scott C., Padilla V., Markides B., Biediger-Friedman L., Crixell S. (2021). O47 Chat with Maya: Assessing the usability of the Texas WIC chatbot. J. Nutr. Educ. Behav..

[33] Hanks A.S., Gunther C., Lillard D., Scharff R.L. (2019). From paper to plastic: Understanding the impact of eWIC on WIC recipient behavior. Food Policy.

[34] Chauvenet C., De Marco M., Barnes C., Ammerman A.S. (2019). WIC recipients in the retail environment: A qualitative study assessing customer experience and satisfaction. J. Acad. Nutr. Diet..

[35] Isaacs S.E., Shriver L., Haldeman L. (2020). Qualitative analysis of maternal barriers and perceptions to participation in a federal supplemental nutrition program in rural appalachian north carolina. J. Appalach. Health.

[36] Weber S.J., Wichelecki J., Chavez N., Bess S., Reese L., Odoms-Young A. (2019). Understanding the factors influencing low-income caregivers’ perceived value of a federal nutrition programme, the Special Supplemental Nutrition Program for Women, Infants and Children (WIC). Public Health Nutr..

[37] WIC Online Ordering Grant. https://www.centerfornutrition.org/wic-online-ordering.

[38] Food Research and Action Center (2019). Making WIC Work Better: Strategies to Reach More Women and Children and Strengthen Benefits Use. https://frac.org/wp-content/uploads/Making-WIC-Work-Better-Exec-Summary-FNL.pdf.

[39] Halverson M.M., Karpyn A. (2022). WIC participants’ perceptions of the cash-value benefit increase during the COVID-19 pandemic. Nutrients.

[40] USDA Proposes Science-Driven Updates to Foods Provided Through WIC. https://www.usda.gov/media/press-releases/2022/11/17/usda-proposes-science-driven-updates-foods-provided-through-wic.

[41] USDA (2022). USDA Proposes Science Driven Updates to Foods Provided through WIC. What They’re Saying. Press Release No. 0256.22. https://www.usda.gov/media/press-releases/2022/11/29/usda-proposes-science-driven-updates-foods-provided-through-wic.

[42] Southern States Generally Have a Higher Share of Infants Participating in WIC. https://www.ers.usda.gov/data-products/chart-gallery/gallery/chart-detail/?chartId=78052.

[43] Ma X., Liu J., Smith M. (2014). WIC participation and breastfeeding in South Carolina: Updates from PRAMS 2009–2010. Matern. Child Health J..

[44] Marshall C., Gavin L., Bish C., Winter A., Williams L., Wesley M., Zhang L. (2013). WIC participation and breastfeeding among White and Black mothers: Data from Mississippi. Matern. Child Health J..

[45] Zhang Q., Zhang J., Park K., Tang C. (2020). Association between usage of an app to redeem prescribed food benefits and redemption behaviors among the Special Supplemental Nutrition Program for Women, Infants, and Children participants: Cross-sectional study. JMIR mHealth uHealth.

[46] Davis R.A., Leavitt H.B., Chau M. (2022). A review of interventions to increase WIC enrollment and participation. J. Community Health.

